# A single-center study of two types of upper kidney preservation surgery for complete duplicated kidney in children

**DOI:** 10.3389/fped.2022.1056349

**Published:** 2022-12-19

**Authors:** Han Chu, Xian-sheng Zhang, Yong-sheng Cao, Qi-fei Deng

**Affiliations:** ^1^ Department of Urology, The First Affiliated Hospital of Anhui Medical University, Anhui, China; ^2^ Department of Urology, Anhui Provincial Children's Hospital Affiliated to Anhui Medical University, Hefei, Anhui, China

**Keywords:** children, duplex collecting system, ureterovesical replantation, ureteral end-to-side anastomosis, ureteral stents

## Abstract

**Objective:**

The objectives of this study were to compare the efficacy, advantages, and disadvantages of insertable ureteral reimplantation (UC group) and ureteral end-to-side anastomosis (UU group) in the treatment of duplicated kidney and summarize the clinical experience in its diagnosis and treatment.

**Methods:**

The current retrospective study enrolled 20 cases with duplicated kidney in Anhui Provincial Children's Hospital from April 2016 to June 2021, including 11 in the UC group and 9 in the UU group. There were 8 boys and 12 girls, with 12 on the left side and 8 on the right side. Meanwhile, there were three cases with urinary tract infection and nine with urinary incontinence. The rest of them were found by B ultrasound during physical examination. The median age of these patients was 33.5 months. Later, preoperative and postoperative renal pelvis separation, ureteral dilation, operation time, and drainage tube indwelling time were compared between the two groups.

**Results:**

There were statistically significant differences in operation time (282 ± 50.55 vs. 176 ± 61.92, *P* = 0.03), drainage time (9.36 ± 5.00 vs. 5.33 ± 1.22, *P* = 0.02), and hospital stay (22.18 ± 5.40 vs. 14.78 ± 5.33, *P* = 0.007) between the two groups. In addition, the degree of hydronephrosis (UC: 1.86 ± 0.93 vs. 1.08 ± 0.77, *P* = 0.00; UU: 1.8 ± 0.95 vs. 0.89 ± 0.60, *P* = 0.02) and ureteral dilatation (UC: 1.57 ± 0.30 vs. 0.72 ± 0.22, *P* = 0.00; UU: 1.47 ± 0.50 vs. 0.88 ± 0.22, *P* = 0.001) were statistically different between the two groups before and after surgery.

**Conclusion:**

Compared with the UC method, the UU method has the advantages of less trauma, faster recovery, and fewer complications. Double J tube or ureter stent placement is beneficial for finding and protecting the lower ureter intraoperatively, without increasing the difficulty in operation, which can also prevent anastomosis or ureteral orifice stenosis.

## Background

Duplex pelvis and ureter malformation is a frequently seen urinary malformation in children, and its incidence rate is 0.8%–1%, with a higher incidence in girls (1). It is classified into complete and incomplete types, with the former being more common than the latter. Vesicoureteral reflux (VUR) is found in 66%–72% of complete duplications and 47% of incomplete duplications; ureteroceles affect 0.025%–0.2% of children in autopsy studies, 80% of ureteroceles are associated with the upper pole ureter of a duplicated system, and 60%–80% are located ectopically (2). The pathology of complete type can be complicated by a ureterocele, ectopia ureteral opening, vesicoureteral reflux, and upper renal dysplasia. Its clinical manifestations include recurrent urinary tract infections (UTIs), urinary incontinence, and abdominal pain (3). Children with duplex pelvis and ureter malformation who have the above clinical manifestations usually require surgical intervention (4). The remedy for duplex pelvis and ureter malformation is no longer single surgical resection, but an individualized remedy primarily based on disease conditions. This study retrospectively analyzed the clinical data of patients undergoing insertable ureteral reimplantation (UC group) and ureteral end-to-side anastomosis (UU group) in our hospital and conducted a single-center comparative study on both renal preserving surgical methods to evaluate their efficacy and understand the effect of double J tube on the operation and postoperative recovery of children.

## Materials and methods

### Clinical data

The present retrospective study was carried out on 20 cases who had a complete duplicated kidney with sparing upper kidney in our hospital from April 2016 to June 2021, including 11 in the UC group and 9 in the UU group.

Inclusion criteria:

(i) Complete duplicated kidney;
(ii) No abnormality of the lower kidney or ureter;
(iii) Upper renal hydronephrosis, and VUR or stenosis of the upper ureter;
(iv) Symptoms of urinary incontinence or UTIs; and
(v) Urinary computerized tomography (CT) and intraudio videoenous urography (IVU) revealing a certain of upper kidneys worth preserving.

Exclusion criteria:

(i) Incomplete duplicated kidney;
(ii) VUR or terminal stenosis in the lower renal ureter; and
(iii) Concurrent ureteropelvic junction obstruction (UPJO) in upper or lower ureter.

There were 8 boys and 12 girls with the median age of 33.5 months enrolled into this study, including 12 cases on the left side and 8 on the right side. Moreover, three cases had urinary tract infection, nine had urinary incontinence, and the rest were found by B ultrasound during physical examination. All cases underwent urological B ultrasound, magnetic resonance urography (MRU), urological CT, IVU, and voiding cysto-urethra urography (VCU) examinations preoperatively. According to the excretion of contrast agent and the morphology displayed by urinary CT and IVU, it was possible to determine whether the duplicated upper kidney should be preserved. All the cases were diagnosed with complete duplicated kidney, with hydronephrosis of the upper kidney and tortuosity of the upper ureter, but with no obvious abnormality in the lower kidney or ureter. One patient had ipsilateral vesicoureteral reflux of the upper ureter (grade I), and two developed an ipsilateral ureteral terminal cyst of the upper ureter. Children with urinary tract infections received anti-infective treatment and underwent surgical intervention after reviewing a normal urine routine. Thereafter, preoperative and postoperative renal pelvis separation, degree of upper ureteral dilation, operation time, and drainage tube indwelling time were compared between two groups.

### Surgical procedure

The two groups of surgeons were in the same treatment team with experienced laparoscopic skills.

**Figure 1:**
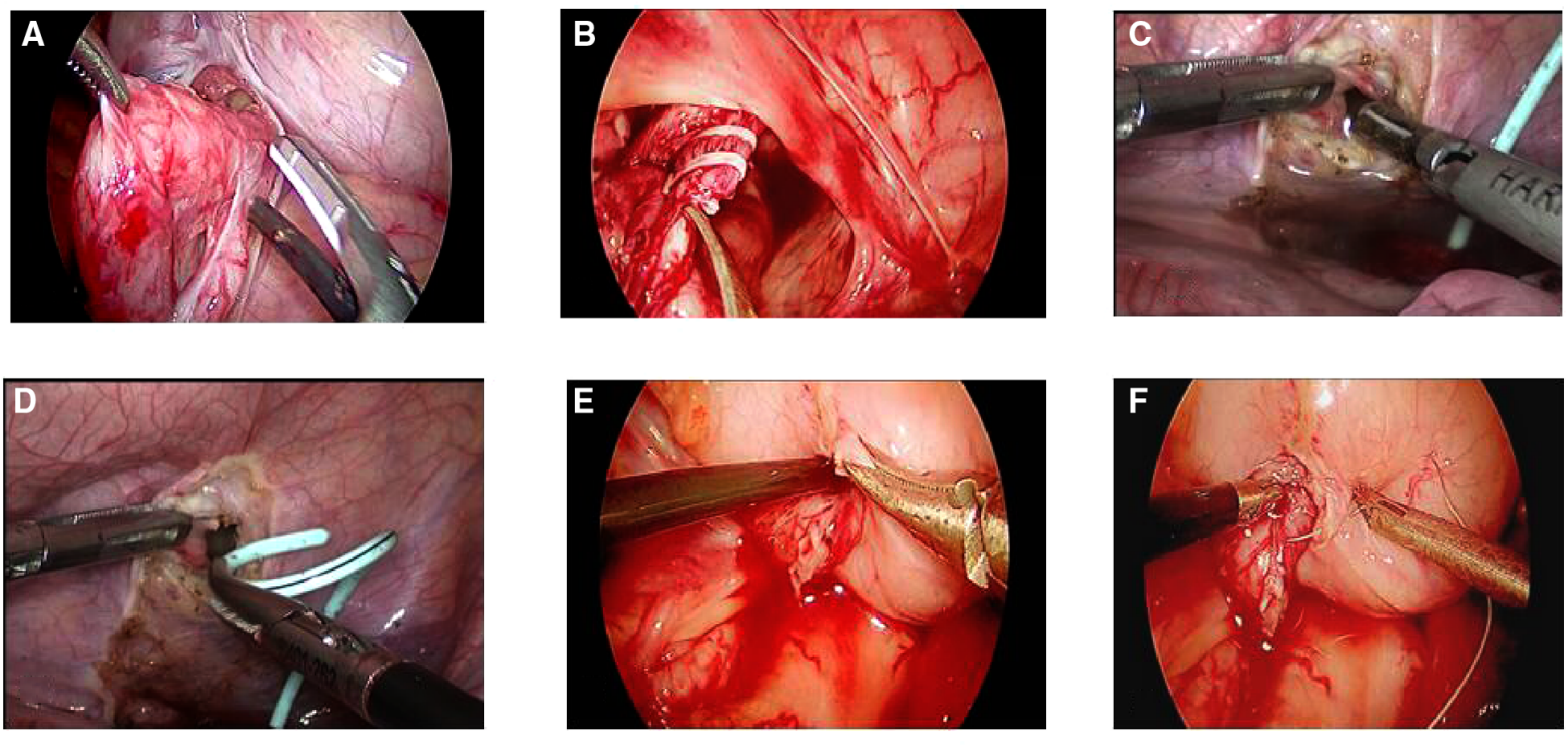
(UC method): (**A,B**) the lower end of the upper renal ureter was exposed, ligated, and dissected (ligated the distal ureter with vesicoureteral reflux or set aside without vesicoureteral reflux). (**C–E**) Double J tube placement into the upper renal ureter, a bladder incision was made above the lower renal ureterovesical junction, and the ureter and the double J tube was inserted into the bladder together. (**F**) The whole bladder layer and ureteral plasma muscle layer were sutured and fixed with an absorbable line.

UC group: After general anesthesia and CO_2_ pneumoperitoneum construction, a small incision was made in the lower margin of the umbilicus, and then a 5-mm trocar was inserted. Thereafter, 3- and 5-mm trocars were inserted into the midline of the clavicle below the eyepiece, respectively. Then, the distal end of the upper renal ureter was exposed, ligated, and dissected. Attention should be paid not to hurt the lower renal ureter. The upper renal ureter was severed near the bladder (the distal ureter was ligated with vesicoureteral reflux or set aside without vesicoureteral reflux), then the double J tube was placed into the upper renal ureter, and a bladder incision was made above the lower renal ureterovesical junction. Subsequently, the ureter was inserted 1–1.5 cm into the bladder, and the whole bladder layer and ureteral muscle layer were sutured and fixed with an absorbable line. A pelvic drainage tube was indwelled and fixed (Figure 1).

**Figure 2:**
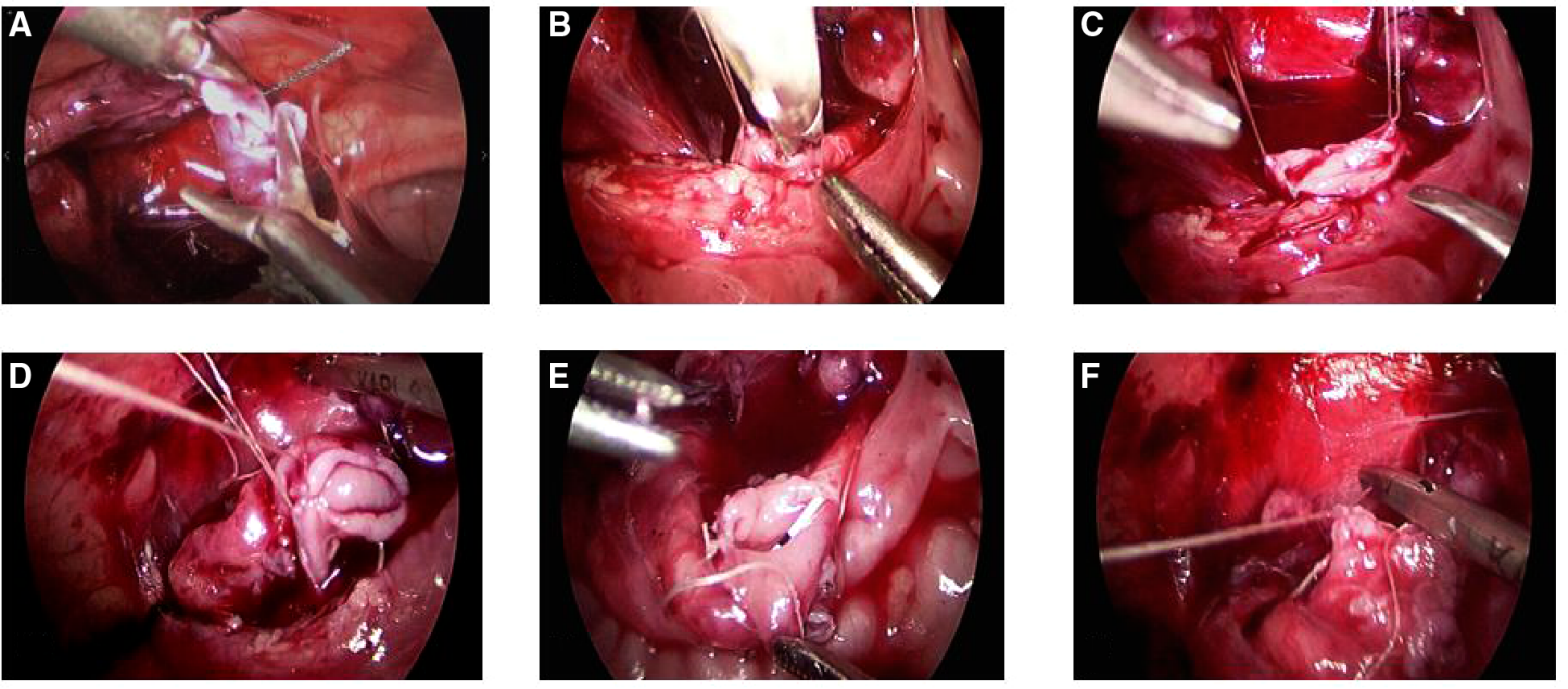
(UU method): (**A**) The upper renal ureter was exposed, severed near the bladder(ligated the distal ureter with vesicoureteral reflux or set aside without vesicoureteral reflux). (**B,C**) the lower renal ureter was suspended and severed longitudinally. (**D**) The anterior side of the ureteral end-to-side anastomosis was sutured with absorbable suture. (**E**) Double J tubes are interleaved into the upper and lower ureter. (**F**): The posterior side of the ureteral end-to-side anastomosis was sutured with absorbable suture.

UU group: After general anesthesia, to observe the ureteral opening in the bladder with a cystoscope, the ureteral stent or double J was placed retrogradely in the lower renal ureter of the affected side, then the cystoscope was removed, and catheterization was retained. After routine disinfection again, a small incision was made in the lower margin of the umbilicus, a 5 mm trocar was inserted, and CO_2_ pneumoperitoneum was constructed. Thereafter, 3 and 5-mm trocars were inserted beneath either side of the umbilical incision. The tortuous upper renal ureter of the affected side was exposed, which was then severed near the bladder (the distal ureter was ligated with vesicoureteral reflux or set aside without vesicoureteral reflux), and the lower renal ureter was found through a double J tube. Subsequently, a longitudinal incision of about 1 cm was made in the lower ureter beside the iliac vessel, while the upper renal ureter was cut diagonally, and the upper-lower ureters (end-to-side) were sutured intermittently with an absorbable line (a double J tube was also placed in the lower renal ureter or crossed into the upper-lower ureter before the anastomosis was closed). A pelvic drainage tube was indwelled and fixed (Figure 2).

Catheters were removed about 10 days after surgery in both groups, and pelvic drainage tubes were removed according to the drainage conditions (the standard for drainage tube removal: <5 ml/day).

### Statistical analysis

SPSS 16.0 software was adopted for performing data collection and analysis. The independent sample *t*-test was employed to compare the operation time, diameter of the ureter, anteroposterior diameter (APD) before and after surgery, length of hospital stay (LOS), and pelvic drainage tube removal time between the two groups. Continuous measurement data on age and follow-up time were represented by medians. In our statistical analysis, *P* < 0.05 was regarded to be of significance.

## Results

Operations were successfully performed in both groups without conversion to laparotomy. For all the children, the double J tubes were removed 1 month after surgery, and there was no case of febrile urinary tract infection (FUTI), anastomotic obstruction, vesicoureteral reflux, residual ureteral syndrome, or abdominal pain and discomfort. Recurrent cysts were not found in the two children with ureteroceles. In only one case, the drainage tube was left for a long period of time, since the standard for removal was not met. After 3 days of tube clamping and ultrasound examination, it was confirmed that there was no urine leakage and no discomfort in the child, and the drainage tube was removed. The patients were followed up for 1, 3, 6, 9, and 12 months after double J tube removal, with the average follow-up time of 20.55 (5–12) months. Hydronephrosis and tortuous ureter were reduced in 17 cases and remained unchanged in 3 cases, with no aggravation in the children. There were statistical differences between the two groups in the operation time (*P* = 0.03), drainage time (*P* = 0.02), LOS (*P* = 0.007), degree of APD (UC: *P* = 0.00, UU: *P* = 0.001), and ureteral dilatation (UC: *P* = 0.00, UU: *P* = 0.02) before and after surgery. The UU method had the shorter operation time, drainage time, and LOS. It was suggested that the long hospital stay in the UC group was related to the long postoperative drainage indwelling time, indicating the greater UC procedure-induced trauma than the UU procedure. There was no significant difference in the degree of preoperative and postoperative hydronephrosis or ureteral dilatation (*P* = 0.88 and *P* = 0.21 preoperatively; *P* = 0.375 and *P* = 0.92 postoperatively) between the two groups (see Table 1 for details).

**Table 1:** Preoperative, intraoperative, and postoperative demographic characteristics.

	UC group		UU group	*P* value		
No.	11		9			
Left	4		6			
Right	7		3			
Boy	5		3			
Girl	6		6			
Median age (months)	31 (7–120)		36 (5–84)			
Operation time (min)	282 ± 50.55		176 ± 61.92			*P* = 0.03
Drainage time (days)	9.36 ± 5.00		5.33 ± 1.22			*P* = 0.02
LOS (days)	22.18 ± 5.40		14.78 ± 5.33			*P* = 0.007
Preoperative APD (cm)	1.86 ± 0.93		1.8 ± 0.95	*P* = 0.88^a^		
Postoperation APD (cm)	1.08 ± 0.77		0.89 ± 0.60	*P* = 0.21^a^		*P* = 0.00^b^, 0.001^c^
Preoperative ureter diameter (cm)	1.57 ± 0.30		1.47 ± 0.50	*P* = 0.38^a^		
Postoperation ureter diameter (cm)	0.72 ± 0.22		0.88 ± 0.22	*P* = 0.92^a^		*P* = 0.00^b^, 0.02^c^
Complication	N		N			
Clavien–Dindo	1^d^		0			

LOS, length of stay; APD, anteroposterior diameter; FUTI, febrile urinary tract infection.

Complication contains: FUTI/anastomotic obstruction/vesicoureteral reflux/residual ureteral syndrome or abdominal pain.

^a^ There was no statistical difference in APD and ureteral diameter between the two groups pre- and postoperation.

^b^ The APD (*P* = 0.00) and ureteral diameter (*P* = 0.00) of UC group were statistically different before and after operation (*P* < 0.05).

^c^ The APD (*P* = 0.001) and ureteral diameter (*P* = 0.02) of UU group were statistically different before and after operation (*P* < 0.05).

^d^ Grade I, long indwelling time of drainage tube.

## Discussion

Duplicated kidney and ureter malformation is usually found due to the clinical manifestations including urinary tract infection, urinary incontinence, and abdominal pain. There are different clinical treatments for complete duplicated kidney and ureter malformation (5). The treatment concepts of complete duplicated kidney mainly include resection and preservation. Resection of the upper kidney and ureter remains the mainstream treatment for nonfunctional or dysplastic upper kidneys. Yin et al*.* (6) found that the proportions of postoperative complications and reoperation were higher in children with <10% upper kidney function who underwent upper renal sparing surgery than those who did not. The renal nuclide examination can be performed to determine the upper and lower semirenal function of duplicated kidney, aiming to make a decision of whether renal preservation therapy should be conducted. This examination cannot be performed in our center because a nuclear medicine department has not been set yet. Based on CT and IVU examinations, the renal cortex thickness of the upper kidney and the secretion of contrast agent were observed to evaluate whether there was any retention value (7). In this study, preoperative imaging examination showed that it was valuable to preserve the upper kidney in all the children.

For functional upper kidneys, kidney-sparing therapy is advocated. In 1998, Bieri et al*.* reported that ipsilateral ureteroureterostomy was an acceptable alternative to common sheath reimplantation in select patients with single ureteral disease in a duplicated system (8). Gerwinn et al. (9) compared the therapeutic effect between laparoscopic ureteroureterostomy (LUU) and common sheath ureteral reimplantation (CSUR) in children with symptomatic duplex kidneys. They found that LUU was a safe and efficacious treatment option for children with duplex kidney anomalies, which might be used as an alternative to CSUR, and LUU induced less trauma than CSUR. Now in children with complete duplication who have normal lower kidneys and ureters and whose upper kidneys have preservation value, ureteral end-to-side anastomosis (UU approach) and insertable ureterovesical reimplantation (UC approach) are the two main methods for complete duplicated kidney treatment. Both the UU and UC approaches can be treated by open, laparoscopic, and robotic methods. The technology of open surgery has been quite mature, which has an exact effect, but can also induce large trauma as well. Laparoscopy is less invasive and easier to expose the distal ureter than open surgery, which can reduce the incidence of ureteral stump syndrome and have a comparable outcome to open surgery (10). Lee et al*.* compared robot-assisted laparoscopic UU with open UU and concluded that the former was a safe and effective alternative to open UU in children with duplicated anomalies. In addition, the operation time and complication rates were comparable between the two methods, with a slightly shorter length of hospital stay in robotic cases (11). Yang et al. (12) compared the DaVinci Xi robot and laparoscope with the UU method in the treatment of distal ureter stenosis and discovered faster postoperative recovery in the robotic group and comparable efficacy and safety than the laparoscopic group. In our country, the DaVinci Xi robot has not yet been popular due to its high costs, and laparoscopic surgery is still the mainstream treatment. Laparoscopic UU has already been adopted for the treatment of complete duplicated kidney (13). Chandrasekharam and Jayaram (14) proposed that ureteral–ureteral reflux (yo–yo reflux) might occur in UU anastomosis, which was more likely to occur in the higher location anastomosis of the ureters than in the lower anastomosis of the lower ureters, possibly resulting in renal infection and renal scar formation. Gerwinn et al*.* (15) suggested that yo–yo reflux was only a theoretical inference, which was not demonstrated. Even in the case of yo–yo reflux, the reflux only existed in the local anastomosis due to the peristaltic transport of the ureter, which did not necessarily reach the renal pelvis, and did not cause repeated renal infection or renal scar formation. There is no unified requirement for the diameter of the upper kidney ureter and the incision size of the lower kidney during the end-to-side anastomosis, and there are also controversies. Generally, it believed that the ureter diameter >2 cm is not recommended for UU treatment, and the incision size of the lower kidney ureter is recommended to be about 1 cm; otherwise, it is not difficult to cause dysperistalsis of the lower ureter and finally leads to hydronephrosis or other complications (12, 15). Abdelhalim et al*.* (16) applied the UU method to treat the duplex pelvis and ureter children with nonfunctional upper kidney or upper ureter diameter ≥2 cm; good results were achieved, and all the children recovered well postoperatively.

We realize that the difficulty of the operation during the laparoscopic UU procedure lies in the incision of the lower renal ureter and end-to-side anastomosis. During the operation, the upper renal ureter should be anastomosed with the lower renal ureter in an oblique plane. Before the lower renal ureter was incised longitudinally, the head and tail of the intended incision position were suspended from the lateral abdominal wall with an absorbable thread (Figures 2B,C), which lowered the mobility of the ureter and facilitated the operation. For the upper renal ureter with a diameter of about 1.5 cm, end-to-side anastomosis was directly performed, and the children recovered well. Our experience for the upper renal ureter with a diameter greater than 1.5 cm is lacking at present, and the UU method is recommended after trimming the upper renal ureter.

In the UC group, due to the opening of the bladder and its voluntary contractions, urine leakage might occur in the early stage of anastomosis, resulting in peritoneal inflammation and the increased exudate. Villanueva (17) suggested that it was unnecessary to open the bladder in the UU method, which thus had a smaller trauma than the UC method. The UC method is prone to bladder spasm or leakage, which may impair the bladder function, especially in children younger than 1 year, and may induce ureteral opening stenosis or vesicoureteral reflux (18). In our center, there was no case of vesicoureteral reflux or ureteral opening stenosis with the UC method. Tang et al*.* (19) proposed that insertable ureterovesical reimplantation after nipple formation at the distal ureteral opening reduced the probability of ureteral reflux and anastomotic stenosis, while the laparoscopic procedure required relatively high techniques. The patients studied in our center did not receive papillary ureterovesical replantation. As a result, its efficacy was not evaluated. Noteworthily, in the UC method, the opening of the bladder should be close to the ipsilateral junction of the lower renal ureter and bladder. According to the physiological and anatomical structure, the upper ureters of female children must be anastomosed with the bladder under the fallopian tube. Otherwise, hydronephrosis may develop when they are pregnant. In both procedures, the emergence of the ureteral stump syndrome should be prevented, and thus the distal ureteral stump should be ligated in children with reflux.

Depending on the surgeon's preferences, the double J tube is placed differently between the two surgical approaches. In UC group, the double J tube was normally placed under the laparoscope, but that was usually placed into the lower ureter retrogradely with the cystoscope first in UU group. The double J tube can also be placed in the lower renal ureter laparoscopically during the UU approach, but it was also cross-placed through the anastomosis (namely, the head end was in the upper renal ureter, while the distal end was in the lower renal ureter) (Figure 2E). Wong and Braga (20) treated duplicated kidney by ipsilateral distal ureteroureterostomy (U-U) with or without ureter stenting. They found that patients with stent implantation had minor complications (two cases of UTI and two of stent displacement). In combination with our surgical experience, the double J tube placed in ureter does not increase the difficulty in the operation or affect the children's postoperative recovery. Consequently, it can serve as a guide and protection for the ureter during the operation, and prevent the end-to-side ureteral anastomosis and new ureteral orifice stenosis after surgery. Because double J tube has no anti-reflux effect, it will cause urine reflux to the renal pelvis and increase the risk of urinary tract infection when the pressure in the bladder increases. Therefore, it is recommended in our center that children with double J tube who have normal urine routine should take 1/3–1/4 of the therapeutic dose of cephalosporin antibiotics before sleep every day, and no FUTI is found in all patients.

## Conclusions

In conclusion, the two surgical methods have their own advantages and disadvantages, and it is essential to choose the appropriate method according to the situation of the children and the surgical experience of the surgeons. The UU procedure is less traumatic, and children can recover faster. For those with experienced laparoscopic operation, insertable ureterovesical reimplantation can be performed after forming the ureteral papillae in order to lower the incidence of vesicoureteral reflux and ureteral orifice stricture. The placement of a double J tube or a ureter stent is beneficial for finding and protecting the ureter during surgery, without increasing the difficulty in operation, and it can also prevent anastomosis (UU surgery) or new ureteral opening stenosis (UC surgery) postoperatively. However, the UU approach can provide the benefits of minimal invasiveness and less complications, making it even more appealing.

## Data Availability

The original contributions presented in the study are included in the article/Supplementary Material, further inquiries can be directed to the corresponding author.

## Limitations of the present study

The limitations in our study are the small series, long hospital stay, and lack of comparison between preoperative and postoperative renal radionuclide imaging; long-term effects should be further followed up.
